# The Impact of an eHealth Portal on Health Care Professionals’ Interaction with Patients: Qualitative Study

**DOI:** 10.2196/jmir.4950

**Published:** 2015-11-24

**Authors:** Anita Das, Arild Faxvaag, Dag Svanæs

**Affiliations:** ^1^ Department of Neuroscience Faculty of Medicine Norwegian University of Science and Technology Trondheim Norway; ^2^ Department of Computer and Information Science Norwegian University of Science and Technology Trondheim Norway

**Keywords:** bariatric surgery, online communication, eHealth, patient, health care, Web 2.0

## Abstract

**Background:**

People who undergo weight loss surgery require a comprehensive treatment program to achieve successful outcomes. eHealth solutions, such as secure online portals, create new opportunities for improved health care delivery and care, but depend on the organizational delivery systems and on the health care professionals providing it. So far, these have received limited attention and the overall adoption of eHealth solutions remains low. In this study, a secure eHealth portal was implemented in a bariatric surgery clinic and offered to their patients. During the study period of 6 months, 60 patients and 5 health care professionals had access. The portal included patient information, self-management tools, and communication features for online dialog with peers and health care providers at the bariatric surgery clinic.

**Objective:**

The aim of this study was to characterize and assess the impact of an eHealth portal on health care professionals’ interaction with patients in bariatric surgery.

**Methods:**

This qualitative case study involved a field study consisting of contextual interviews at the clinic involving observing and speaking with personnel in their actual work environment. Semi-structured in-depth interviews were conducted with health care professionals who interacted with patients through the portal. Analysis of the collected material was done inductively using thematic analysis.

**Results:**

The analysis revealed two main dimensions of using an eHealth portal in bariatric surgery: the transparency it represents and the responsibility that follows by providing it. The professionals reported the eHealth portal as (1) a source of information, (2) a gateway to approach and facilitate the patients, (3) a medium for irrevocable postings, (4) a channel that exposes responsibility and competence, and (5) a tool in the clinic.

**Conclusions:**

By providing an eHealth portal to patients in a bariatric surgery program, health care professionals can observe patients’ writings and revelations thereby capturing patient challenges and acting and implementing measures. Interacting with patients through the portal can prevent dropouts and deterioration of patients’ health. However, professionals report on organizational challenges and personal constraints related to communicating with patients in writing online. Further development of guidelines and education of health care professionals about how to handle, prioritize, communicate, and facilitate patients online is required in addition to increased attention to the organizational infrastructures and incentives for enabling such solutions in health care.

## Introduction

Given the limited time for face-to-face consultations, health care professionals and patients experience considerable challenges in setting priorities to address clinical concerns. New approaches to organize and deliver health services are being explored and eHealth technologies are one of the key elements to address this. Promises about improved cost-effectiveness by the use of such may reduce the pressure on the health care system and improve the quality of care for the recipients [1-3].

### Weight Loss Surgery

The number of people suffering from obesity and obesity-related comorbidities has increased significantly the last couple of decades [4,5] entailing enormous economic and health costs [6]. The effects of obesity are reversible and have led to a rising demand for weight loss interventions [4,7-9]. Bariatric surgery (weight loss surgery) is currently one of the most effective interventions to produce initial weight reduction [7,10] and the number of performed surgeries has increased dramatically over the past decade [7,8]. Most surgeries nowadays are performed with short hospital stays. A number of aspects prove that this is both cost-effective and considered beneficial for the individual [9,11,12]. Bariatric surgery procedures are no exception because patients are procedurally discharged a couple of days after surgery if no complications have incurred [11]. Accordingly, the outcomes depend on the patients’ adherence to recommended treatment regimens and on their abilities for self-care management.

### Challenges Related to Bariatric Surgery

Even though bariatric surgery is one of the most effective interventions to produce initial weight reduction, there are many challenges related to the treatment. Patients commonly experience difficulties, particularly the first period after surgery because of the immediate impact of the surgical procedure on their physical well-being. The purpose of the surgery is to restrict food intake and involves removing and bypassing parts of the intestine. The operation contributes to reduced absorption, leading to poor digestion and reduced nutritional uptake. As a consequence, the patients must follow a particular dietary regimen and, in some cases, are required to take lifelong vitamin supplements to prevent nutritional deficiencies with severe outcomes [13-16].

The surgery alone does not suffice to achieve successful outcomes; the patients need to change their lifestyle, addressing dietary habits and physical activity in order to accomplish results [17,18]. Research shows that bariatric surgery patients experience challenges after some time because the recommended lifestyle and behavior changes are difficult to maintain [19,20] and many patients regain weight [20-24]. The underlying reasons for weight regain are multifactorial: the causative factors are patient-related (mental health and behavior) and surgery-related (anatomical alterations and complications) [25]. Weight regain is an important public health issue with significant consequences to the patient as to the recurrence of obesity-related comorbidities and to the health care system due to the economic costs of obesity and societal impacts of recalcitrant obesity. In an effort to manage and prevent weight regain, an organized and systematic approach is essential [25].

Most bariatric surgery clinics offer some kind of follow-up to their patients; these are typically telephone conversations, individual face-to-face consultations, or group-based meetings. However, this group of patients commonly experience stigma and shame [26,27], and restrain from making contact with health care professionals trough traditional means, such as by telephone or meeting in person [28]. In worst-case scenarios, this might result in fatal consequences because complications or other challenges might not be acknowledged and adequately handled. Therefore, the need to facilitate bariatric surgery patients in connection to their treatment program is critical to provide sufficient health care delivery and clinical care to this patient group. Toussi et al [20] pointed out that having more contact with patients and requiring adherence to behavioral changes, especially with respect to exercise and dietary restrictions, may improve the long-term outcomes for bariatric procedures.

### eHealth Portals in Health Care

eHealth solutions, such as secure online portals, hold great potential if offered to patients in conjunction with their treatment program because they create new opportunities for improving health care delivery and follow-up of clinical care [1,29]. eHealth portals offer a number of potential benefits to providers, including administrative efficiencies, improved responsiveness to patients’ needs, decreased utilization of health services, more effective care, and cost savings [30]. Despite the potential advantages, the adoption of eHealth solutions and portals has been low [30,31]. The success depends on the degree of acceptance by its users, where health care professionals are key stakeholders to adoption and use [32,33]. A number of barriers to adoption have been identified, such as concerns about costs, added workload and workflow demands, technology literacy, liability issues, and confidentiality and privacy risks [30,34,35]. To our knowledge, few studies have explored Internet-based tools such as eHealth portals in bariatric surgery. A number of studies have been done in other areas of chronic disease management, such as in diabetes care, chronic obstructive pulmonary disease (COPD), and asthma [36-41]. eHealth portals in diabetes and COPD show that access to information and support via online patient-centered tools improves patient engagement and health outcomes, but there are unclear results when it comes to the effectiveness of follow-up [37,38]. In diabetes care, studies show that providers often are reluctant to adopt these technologies due to lack of knowledge about the Internet or information technology systems [40,42]. There are few, if any, studies exploring health care providers’ perspectives on the use of eHealth portals in bariatric surgery. Because the impact and success of such solutions depends on the organizational delivery systems and the professionals’ acceptance and adaptation of the solutions, the need to explore their views is important.

### Objective

The objective of this study was to characterize and assess the impact of an eHealth portal on health care professionals’ interaction with bariatric surgery patients. The aim was to develop a better understanding and insights relevant for using such solutions for health care delivery and care in bariatric surgery programs.

## Methods

### Study Setting

In this research project, an eHealth portal for bariatric surgery patients was established in 2011 in collaboration with a bariatric surgery clinic in Norway. The portal was developed through a human-centered development process [43] and according to the security and privacy concerns that are required for such solutions in Norway. Access to the portal required log-on procedures with username, password, and entering of a one-time personal identification number (PIN) sent to the user’s mobile phone. The features of the eHealth portal included:

1. Patient information (eg, validated information about the surgery, pre- and postsurgical recommendations, food and diet, nutritional facts, lifestyle recommendations, physical activity)
2. Self-management tools (eg, personal diary, calendar, reminders via short message service [SMS] text messaging)
3. Communication features (for dialog with peers and providers)
4. Online discussion forum
5. Personal messaging

**Figure 1:**
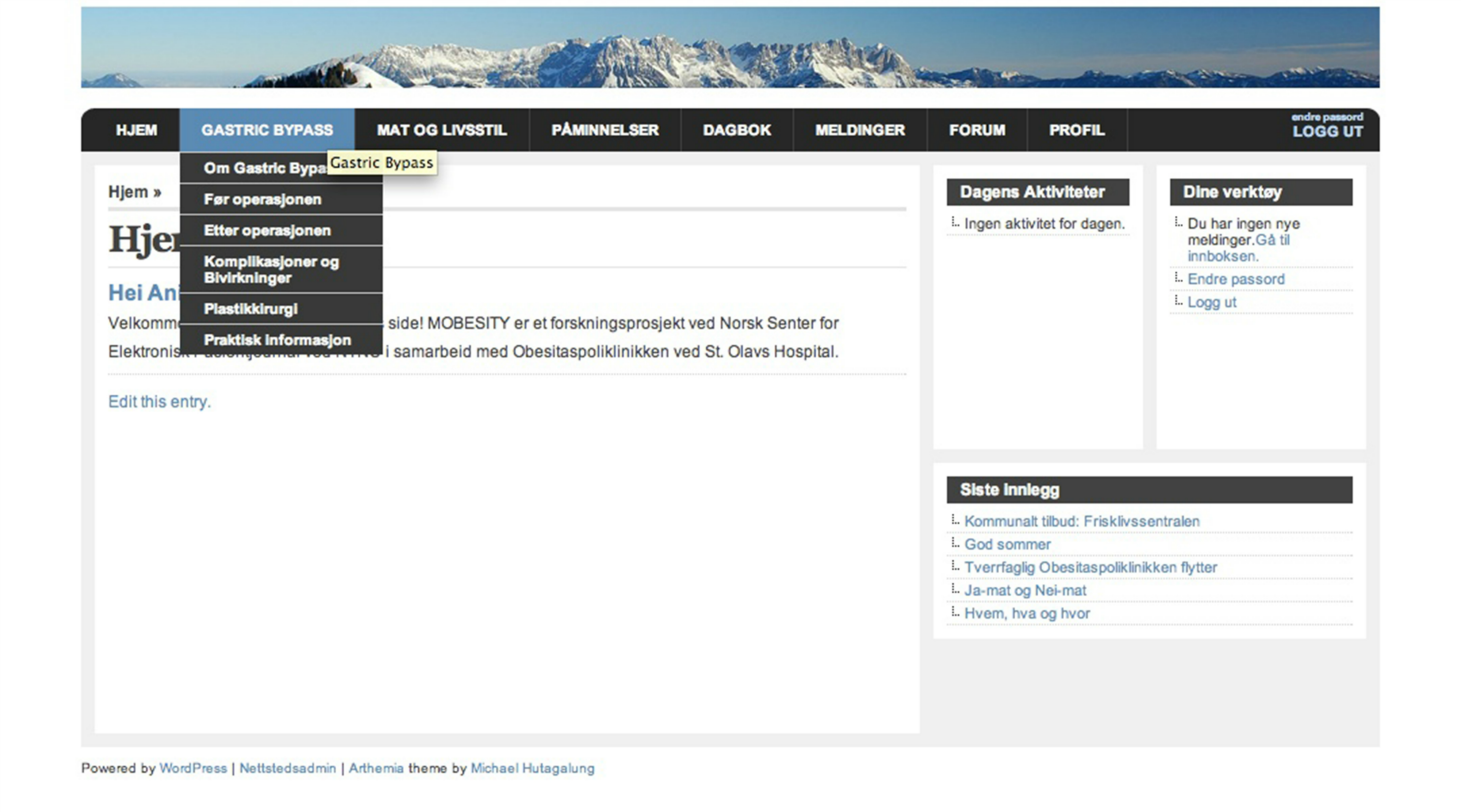
Screenshot of the eHealth portal.

The eHealth portal (Figure 1) was implemented in the bariatric surgery clinic, where 5 health care professionals (all women; 2 nurses, 1 clinical dietician, 1 psychiatric nurse, and 1 administrative leader) at the clinic received access to facilitate the patients and respond to their requests. In addition, one person from the research team, educated in nursing, had the overall responsibility to moderate the forum and could comment on postings that were within her field of competence. The patients received access to the eHealth portal for approximately 6 months. In total, 60 bariatric surgery patients (75%, 45/60 women and 25%, 15/60 men) received access and 80% (48/60) of them logged on to the system one time or more. The study was approved by the Regional Ethics Committee [44] and by the Norwegian Social Science Data Service [45]. All participants provided written informed consent when included to the study.

### Data Collection and Analysis

Data collection involved a field study and in-depth interviews with health care professionals. The field study was conducted at the clinic, consisting of contextual interviews with professionals at the clinic during the 6-month study period. Such interviews involve observing the people in their actual work environment and speaking with them about their work and actions [46]. The contextual interviews typically lasted 20 to 60 minutes, were informal, and notes were taken. At the end of the study period, the 5 health care professionals were requested to give in-depth interviews [47], but not all could participate. Semi-structured in-depth interviews were conducted with 3 health care professionals. The interviews were conducted in Norwegian, lasted 1 hour each, were sound recorded, and transcribed verbatim before analysis. The two first authors conducted the analysis, which was done inductively using thematic analysis, and used English terms and concepts. HyperRESEARCH software was used to facilitate the process, involving a stepwise process in which both researchers reviewed the material and created codes individually. Next, the codes were collated and concepts were generated in a mutual process. These were compared, contrasted, and discussed in light of relevant literature and theory, and the final themes were achieved via consensus. The quotes in this paper are translated from Norwegian to English and the names reported are pseudonyms.

## Results

The analysis revealed two main dimensions of using an eHealth portal in bariatric surgery: the transparency it represented and the responsibility that followed by providing it. The personnel reported the eHealth portal as (1) a source of information, (2) a gateway to approach and facilitate the patients, (3) a medium for irrevocable postings, (4) a channel to expose responsibility and competence, and (5) a tool in the clinic.

### The eHealth Portal as a Source of Information

The health care professionals reported the eHealth portal to be a source of information in regards to gaining awareness about the unique challenges of the individual patients and as a learning source about the group of bariatric surgery patients. With access to the portal, the patients could write and post whatever they wanted, whenever they wanted. Most of their postings were stories and narrations about personal experiences; they shared thoughts about daily ups-and-downs, often without specific questions requiring attention. Some patients used the online forum as an arena to post their “personal diaries.” The professionals reported that the length of the postings and/or the number of threads related to a particular topic could signify a problem that needed attention; therefore, they read most postings even though they were not addressed to them in particular. “Linda” described “reading between the lines” to identify if anything was out of the ordinary: “Even though it’s there as part of a diary, and there is not a single [direct] question there, you understand that something isn’t how it should be.” During the field study, we observed how she handled such postings: if she considered that the patient needed facilitation by the clinic, she would approach the patient by sending a personal message through the portal to identify if there were issues that needed further investigation. All professionals who had access to the portal reported that they were surprised about the vast and rich amount of information about the patients that became available through the portal. Some issues and themes were recurring, posted by several patients, signifying what information this group of patients searched for and needed:

We have learned a lot as well. So we need this type of patient contact.

Linda

The patients posted a great number of questions; some were meant for their peers, others were addressed to the health care professionals. The professionals reported becoming aware of issues they previously had not considered significant. They knew that the patients experienced challenges in adjusting their lifestyle, but they were not aware of how complicated this turned out to be. The insights that became evident by reading the postings concerned the patients’ unique experiences postsurgery, the psychosocial aspects that came to pass, and the enormous challenges they experienced related to the new lifestyle and diet. These understandings benefited the patient group:

We can capture the information they write. In addition, we can learn a bit more about how to facilitate the patients.

Bente

The information they attained was important for their occupational behavior, knowing what to emphasize in contact with the patients:

In relation to the need of feeling cared for as patients, it is probably useful. And it’s educational for us as well. Because learning goes both ways.

Fride

The knowledge gained was further enabled to customize the patient information and contents in their patient education program.

### The eHealth Portal as a Gateway to Approach and Facilitate the Patients

The eHealth portal worked as a lowered threshold solution for the patients to seek advice, guidance, and help, and as a gateway for the health care professionals to approach and facilitate the patients.

#### Lowered Threshold Solution

By following the patients’ writings, the professionals got an overall impression about the patients’ daily lives as opposed to the selected issues they were presented during time-limited face-to-face consultations:

But it’s obvious that one can capture things in the portal that I cannot capture during a consultation.

Fride

In the patients’ online writing, their information was described in greater detail compared to oral contexts:

You get more information about them here [online] than on the phone.

Bente

This was considered to be relevant in order to identify patient symptoms and needs: “...because, in the portal they are more laid back and at home...and they are closer to what is relevant for them there and then.”

They found that some patients had difficulties in revealing their actual problems in face-to-face settings:

Those who come for consultation and sit in that chair and talk to the person in white coat, I don’t think it’s always that easy for the patient to come with his or her request to me.

Fride

In the field study, we observed that the patient consultations often ran overtime and other patients were kept waiting. The professionals described that some patients’ required significant time during the consultations because they needed time to feel confident and had complex needs. However, because other patients were waiting for their turn, the personnel had to end the consultations even though they knew that the patients had more on their mind. The professionals reported that factors such as time constraints, shame, and fear of stigma could influence the oral dialog and thereby restrict what the patients were comfortable in sharing in face-to-face settings. These issues were not as prevalent for the patients when communicating online. The personnel quickly learned that some patients found it easier to take contact with the clinic through the eHealth portal: “Yes...they give their notice here instead of calling...” Also, they observed that some preferred to express themselves in writing via the portal and, therefore, it became a lowered threshold solution: “...they are at home, it’s easier to send a message online than to call, and that’s why we get so many questions.”

#### Dropouts

When undergoing bariatric surgery, the patients were offered a 5-year follow-up program at the clinic consisting of a combination of group-based and individual outpatient consultations. These consultations occur at specific intervals after surgery: at 3 months, 6 months, and 12 months after surgery, and at yearly intervals for the following 4 years. The health care professionals reported that a number of patients failed to show up to these scheduled (face-to-face) consultations, something we also observed in the field. Even though they rescheduled the appointments, sent letters, and tried to achieve contact by phone, some patients still did not show up, thereby dropping out of the follow-up program. This represented a challenge for the clinic because they were left with no data about the cause or how these patients coped after the surgery. “Linda” observed patients having difficulties in achieving their expected outcomes:

The operation, it is kind of their last chance. And if they don’t succeed with that either...they say that they think, “Oh my God, now I got this operation costing 100,000 NOK, and all that help and follow-up, and still it doesn’t work”.

The personnel stated that several patients refrained from taking contact with the clinic by traditional means when necessary because of shame:

If this can be that place where those who struggle and who do not want to show up in person because of shame...because it is shameful not to be able to make it [lose weight], right? One had great expectations and then it did not go as planned...If we can get hold of them through this, then it’s really good. Because we want everyone to succeed.

Fride

As a consequence, the clinic could not follow up and provide health care to patients they perceived needed it:

In reality, I think that there are more people that struggle than those who say they do. Who need help, and yes...they are ashamed.

Linda

They detected that some of those who excluded themselves from the traditional follow-up program were active on the website:

And not everybody who are in here [the portal] makes contact with us by phone...because not everybody, I don’t think that everyone that are in here would take contact with us otherwise.

Bente

“Linda” discovered that one of their patients failed to show up to her scheduled consultations, but posted considerably on the forum. By following her postings, she understood that the patient needed help and initiated contact through the portal. They communicated in private messaging and identified that she needed additional medical investigations and scheduled her for further follow-up to the endocrinologist at the clinic. Later, this patient expressed gratitude about receiving the care she needed due to the portal because she would not have taken contact with the clinic directly. The portal became an important asset as it represented an additional approach for the professionals to reach out to the patients:

I think that this can be, if we are going to [continue] using it, then this might be a place where we can get hold of them. The people who do not dear to take contact...yes, or who are to shameful to show up at the traditional programs we offer, to meet in person.

Fride

### The eHealth Portal as a Medium for Irrevocable Postings

Interacting with patients in writing online was a new way of communicating and represented other aspects than in an oral dialog. “Fride” reported that this signified uncertainty about how to deal with this new kind of interaction:

I have chosen to read what I have found to be related to my area of competence, and I think that has been okay. Occasionally I have felt that some have disclosed themselves. And I don’t know if that is okay.

“Bente” expressed concerns about the degree of self-disclosure she observed: “They expose themselves too much for the others that are in and have access to read...” The personnel were concerned about what the patients exposed online and were equally apprehensive about their own postings:

It’s just that you have to consider that this can be used against you later in some way, it remains there.

Linda

This was a shared understanding among all the professionals. The awareness about how to communicate online became particularly significant when their posting would be available to many people:

It is about practice—to practice to write short and concise, and dare to be...not vague. At least I am very afraid of writing to concluding, particularly when I am in such a forum, when it will be standing there written. It gets a lot of readers and you try to ensure that what you write is correct.

Fride

When online, nonverbal cues, such as body language, tone of voice, and gaze, that were present in face-to-face conversations disappeared. “Fride” contrasted online communication with a face-to-face dialog, where she continuously would assess whether the patient actually understood what she said and the information she provided. When communicating in writing she had to be particularly aware in order to avoid misinterpretations: “And what I said before, that you have to be sure about that what you say is correct, and that it cannot be understood differently.”

“Linda” shared this understanding:

It’s okay, but you have to consider what you say, when it’s written...I have to be aware about how I articulate myself. It’s almost like when you get an SMS from someone, and “What!? Bad mood today, or what is it?” If I talk to them (patients) on the phone for instance, I hear if someone misunderstands something. Yes, and that you don’t here...have to think carefully, can’t just [write].

She had become used to communicating with family and friends in writing by using SMS text messaging and social media forums, and stated that this transition needed to be undertaken in the professional sphere as well: “Thus, there is something about getting used to communicate this way, and you are in your personal life.” All assumed that with time and practice the clinic would adapt to communicating online with their patients.

### The eHealth Portal as a Channel to Expose Responsibility and Competence

According to the professionals, the questions they received online differed from the ones they traditionally received in oral contexts: “Maybe more specific in the portal. And maybe it is those who are interested, or who try [who ask].” The patients’ access to other information sources seemed to have an impact:

But then the questions here, it’s clearly that the questions that have been posted, those are from patients that have read all the information that is available here [in the portal], and they have also talked to others that have undergone surgery.

Also, the patients’ context when articulating the questions influenced the topics:

Because here they are at home in peace and quiet, and can use—can get information from other arenas as well obviously...The questions have not only been experiential.

Linda

The personnel reported being unprepared for the advanced level of questions they would receive: “Thus, the questions have been really good, often so advanced that we have been required to speak with a specialist.” This represented a challenge for the level of expertise required to provide an appropriate response: “...so there have been many questions that I have not been able to answer, have needed to talk to the specialist.” They could not refer the patient to another professional through the portal because not everyone at the clinic had access. Neither could they ignore the patients’ requests because the presence of unanswered questions could create an impression about not doing their job. As a result, it became necessary to provide high quality answers to the patients’ posts. In cases when the personnel having portal access could not respond themselves, they made contact with other professionals at the clinic, such as the physician, physiotherapist, pharmacologist, surgeon, and endocrinologist, to get quality assured information for redistribution to the patients. The fact that they needed to make contact with other professionals became more obvious when using the portal compared to an oral context:

Now we get quite some calls about that they have pain or...We can’t give the diagnosis [stating her profession] over the phone. And I couldn’t have done that here either.

Bente

When delivering the response in writing, the caregivers felt obliged to take contact with others to ensure a qualified response:

Like “why can’t they take NSAIDs?” That resulted in that our pharmacologist didn’t want to answer, needed a statement from the chief over there.

Linda

“Linda” explained that the activities triggered by this one question required considerable efforts: the process required resources in regards to have expertise in knowing the right addressee, time effort to contact them, have them write a statement, and get the information validated before they could finally post the statement online. In the field study, we observed that the process could take quite some time and effort, which verified the personnel’s experiences. Even though the patients’ requests were specific, the answers from the professionals would not necessarily correspond in level of detail because they delivered an answer based on the information they had available there and then:

When you are in a face-to-face consultation, you have access to much more medical information and about the patients’ history, and you aren’t supposed to give advice without knowing, without knowing the underlying cause. So it’s, call it whatever you want to, but it’s a weakness as well, and then you have to give more general advice, less specific advice, because you don’t know.

Fride

In the field study, we observed that some of the professionals would search the electronic patient record and look up test results if necessary to answer the patients’ requests as best as possible. But the professionals experienced that the online communication had its limitation in cases where they found it necessary to go deeper into the matters to provide sufficient help:

Particularly those who have posted a lot, then it’s preferable that you have read what they have posted before, and not only answer the question. Like the one I just answered, I think it’s a lot, and then there is no use to just answer the last one there, then it’s better to get them to come to a consultation when [you understand] it’s complex.

Linda

Therefore, in some instances, the patients’ postings worked as triggers for further communication, occasionally leading to face-to-face consultations.

### The eHealth Portal as a Tool in the Clinic

In the beginning, when introducing the portal in the clinic, the professionals expected that it would become an integrated tool in their daily occupational practice. They talked about their intentions for using the portal in peace and quiet, focusing on the patients’ posts, and responding to their requests. They assumed that the opportunity to communicate with the patients in an asynchronous manner would give them more flexibility in when to do the work, but the reality turned out to be different from expected and factors such as normal work routines, time constraints, and prioritizations became evident in the daily clinical practice:

Then I can sit down whenever I have time, but on the other hand, I probably have shown that I don’t have the time, or do other stuff, right? So you need to get accustomed to use it.

Bente

The professionals described their work routines to be hasted, characterized by fully booked calendars with appointments and patient consultations, and often interrupted by unexpected telephone calls and other emergent tasks. Thus, their intentions failed to materialize:

It is just that the days are filled with patient lists, and suddenly it is 4 o’clock, and then you are off to home. We haven’t organized the time for it, and we should have. It hasn’t been a priority because when a patient is physically here, then you have to attend to him. If the phone rings, you have to pick up. And then this is what we postpone to use. Unfortunately.

Linda

Enabling a tool that the personnel were unfamiliar with proved to be a restraining factor in getting it integrated into their daily work routines. Using the portal became an extra task in addition to their current duties, which we observed that they prioritized to complete:

It is the time pressure we have at work, we don’t have time for anything. I have to put everything aside, and when I get time I have to catch up [the other work]. So one can say that it has to do with priorities.

Bente

The lack of incentives became prevalent when using the eHealth portal:

It does not give us any incomes because we got feedback about that from our boss that if it does not give us any incomes...we have to register it some way. Because our leader go in and check how many patients we have every day. And then surely you get feedback if you haven’t reported any patients, then you would have gotten some questions.

Bente

It was difficult to justify using the portal when they knew that their work was evaluated based on other criteria:

To be honest, this has not been something I could prioritize. You prioritize those that are on your patient list. Those are the ones you are counted for...how many notes [in the electronic patient record] that are in progress and incomplete and so on. That is something my leaders go inn and check. So that is what you are counted for.

Fride

The organizational infrastructures and economic incomes that the professionals perceived to be important for getting such a tool integrated into their current work routines were nonexistent at the time of this study. These were reported by the personnel to limit portal use and redeem the opportunities it presented.

## Discussion

### Principal Findings

The findings suggest that health care professionals experience a number of benefits from interacting with bariatric surgery patients through a secure eHealth portal while it also poses a distinct set of challenges. The two dimensions of transparency and responsibility that follows by providing an eHealth portal to this patient group became decisive for how the professionals enabled the portal. The transparency to both the patients’ lives and the professionals’ online actions influenced the professionals’ roles and responsibilities toward the patients. The success of implementing such a portal into bariatric surgery care appears to depend on how confident the professionals are in communicating in writing and using online tools as well as organizational infrastructures and incentives. Yet, such online communication portals may place greater demands on the caregivers because it appears to be a solution that the patients both prefer and benefit from using. Traditional communication arenas between bariatric surgery patients and their health care providers seem to have their shortcomings. Thus, professionals must learn how to communicate online and enable eHealth tools as a complement to traditional care for this patient group in order to follow up and facilitate patients in need and consequently enhance patients’ outcomes after treatment.

### Transparency

The eHealth portal provides transparency to the patients’ daily life, their challenges, and their needs, and it became an information source about the patient group. The narratives that patients create and share outside the constraints of time-limited consultations can help professionals develop a more comprehensive view of the situation of their patients, thereby enabling them to individualize the care to the patient’s particular needs. But the transparency goes both ways: an eHealth portal that make the patients’ requests and the health care professionals’ postings available for all to read makes professionals spend more time in preparing comprehensive, thought-through answers compared to communicating in oral contexts. This is a fact that is important to consider when introducing additional tasks for the personnel. Given the fact that their postings would remain online and the fear of publishing information that can be perceived as incorrect, results in the professionals acting carefully and deliberately in their online acts and written communication. Also, each health care professional’s competence becomes evident when using a written communication form, resulting in that they become particularly aware about what they are eligible and comfortable on posting.

### Responsibility

The online portal represents responsibility to follow up and provide high quality health care to the patients. This become particularly evident for following up the patients’ postings because these signalize the professionals’ work; if they do not respond, this can signify poor quality and work. The responsibility can be seen at two levels: with the competence and skills to identify the patients’ challenges and needs, the professionals are obliged to act and implement measures accordingly. On the second level, the responsibility to provide correct and quality assured information becomes evident when communicating online in writing; it becomes an absolute of no discussion when it stands in text. The fact that the professionals “monitor” the patients by accessing their writings and narrations means that they can identify if and when patients experience signs and symptoms of health deterioration that need professional follow-up and care. Given that the professionals, based on their clinical expertise, can identify patient symptoms and needs at an early stage, makes them responsible to act and implement measures accordingly. The prevention of health deterioration can have great impact on both the patients’ health status and quality of life, and to society as a whole considering the health care expenses of treatment costs and hospitalizations.

### Implications

Bariatric surgery is often a “last chance” solution to patients who have tried and failed various approaches to achieve weight reduction, which leaves them with unrealistic expectations toward the outcomes of surgery [48,49]. The informants report that patients’ inadequate adherence to the follow-up program were due to unsuccessful outcomes and shame, resulting in restraints in making contact with the clinic when in need and dropouts. These findings correspond with earlier research that show that inadequate adherence to follow-up programs in bariatric surgery is associated with poor weight loss and maintenance, poorer control of obesity-related comorbidities, and the development of postoperative complications [50]. Attrition to bariatric surgery aftercare and weight loss intervention programs is associated with greater presurgical weight, psychological and behavioral patient factors, processes associated with the treatment, and greater travel distance to the follow-up center [50,51]. The portal proves to be a possible gateway for the professionals to communicate and interact with patients, particularly as a channel to a subgroup of patients who for various reasons do not use traditional communication forms currently in use at the clinic and would have been lost to follow-up. Bariatric surgery patients report that they experience difficulties in communicating with professionals in face-to-face meetings [28]. This underlines the need to offer new solutions. The personnel report that some patients prefer to communicate online rather than face-to-face, which implies that they experienced a benefit of using such an eHealth portal. For those who reject participation in the traditional aftercare program, eHealth portals for online communication can be a substitute and be valuable for addressing clinical needs and care. Adherence to scheduled visits (and compliance to recommended rules) predicts success of bariatric surgery [52], where health care professionals can use eHealth portals in communicating and promoting recommended postsurgical regimens. This might be an additional approach or even a substitute for face-to-face visits to selected patients. Better contact between health care providers and patients may improve the long-term outcomes for bariatric procedures [20]; this study has shown that an eHealth portal can be one approach to achieve this.

Despite the potential advantages of using the eHealth portal, the professionals report a number of organizational challenges, such as time constraints, busy working hours, and lack of incentives as underpinnings for their work. These findings are similar to the ones of Hanberger et al [53] who found that practitioners in diabetes care had a hard time starting to make use of an eHealth portal in their practice due to obstacles such as deep-routed working habits and too many working tasks. Enabling and using the portal was more time consuming than anticipated, a finding that is opposed to previous envisions about more efficient use of clinical time by the use of Web-based tools [54]. The professionals had difficulties in justifying the use of a work tool that did not give the clinic income because, in the end, their occupational behavior depends on giving the clinic sufficient earnings. The lack of incentives drives the prioritization of the personnel’s activities and, for increased adoption and use of technology, incentives at both the individual level and organizational level should be considered. At the individual level, remuneration for work efforts can be either financial (eg, reimbursement for activity) or nonfinancial (eg, workload credit for activity) [55]. When introducing a personal health record at the Department of Veterans Affairs, a workload code for secure messaging was implemented to enable workload credit for secure messaging activity, providing incentives at the individual level to foster increased adoption and use of the technology [55].

### Implications for Practice

The findings of this study have demonstrated the feasibility of an eHealth portal for patient care and communication in bariatric surgery, which provides both clinical benefits and challenges. The health care professionals imply that an eHealth portal has great potential and impact in bariatric surgery, but that there are a number of aspects that need to be addressed in order to take full advantage of the benefits. A portal for communicating and interacting with bariatric surgery patients can be a useful complement for most patients, but for selected patients it might be a substitute to traditional postsurgery care. Even though the practitioners are motivated to use the new solution, the fact that they are evaluated by their economic income to the clinic makes them prioritize their work accordingly and the necessity to implement incentives is therefore crucial.

Based on these findings, we present some practical implications that need to be considered when introducing and implementing eHealth portals into clinical practice:

1. Establishment of clinical rationale. Define why and for what purpose the eHealth portal is implemented. What are the major motivations and how should these be communicated to the personnel?
2. Clinical skills and competences. Identify if the personnel have sufficient competencies to identify patients’ symptoms and needs. Are other competencies or skills than those currently available required?
3. Decision support and multidisciplinary team. Assess whether the personnel who will facilitate the patients have sufficient decision support. Do they have a multidisciplinary team available for questions?
4. Individual motivation. Explore the personnel’s individual motivation. Are the personnel motivated to use the eHealth tools? Are they satisfied with the information, training, etc, they have received in order to enable the solution in an efficient manner?
5. Communication skills. Identify the personnel’s competences and experiences with communicating in writing/online. Are the personnel comfortable in communicating in writing? If not, do they need practice or education?
6. Organizational infrastructures. Identify barriers to enable the technology. Do the personnel have time and resources to use the technology? Do they have access to sufficient infrastructures (eg, computers, Internet) and dedicated time when they can use the technology?
7. Clinical workflow. Identify how enabling of the new technology corresponds with the established workflow at the clinic. Which adjustments are required for satisfying integration between current and new tasks?
8. Incentives. Identify which incentives are required for enabling the technology. Is it necessary with economic incentives? Does it require incentives at the individual or organizational level, or both?

### Study Limitations and Future Work

This study is limited due to its qualitative approach, restricted to a case study, and the results cannot be generalized. The results might be different if other informants were involved, a different patient population, another Web portal, or setting was studied.

In this project, the patients had no restrictions about length, topic, or timing for their postings. Because our findings show that the professionals experienced that the time and competence required for handling the postings were significant, this suggests that more structured forms of communication should be investigated in future projects: patients can fill-in predefined categories or answer a particular set of questions. The need to investigate which categories and contents these should include are subject for future investigations. Also, further studies considering quantitative measures and cost-efficiency studies are required when it comes to eHealth portals in bariatric surgery. Our study reveals that communicating with patients and facilitating them online requires certain clinical skills and competences to capture their symptoms and needs. This underlines that not just anyone can be a moderator and recipient to patient requests, but that it requires particular health education in order for the patients’ to be adequately handled. Also, skills in communicating in writing with patients are required when providing such eHealth solutions. The need to acknowledge that these are required competences and educate professionals about how to communicate and interact with patients online is an underestimated issue that needs further attention.

This study revealed a number of aspects that are not directly evident when introducing eHealth portals, but that are extremely important for the tools to be appropriately implemented and adopted in bariatric surgery practices. When considering the use of an eHealth portal in clinical care, the motivation and clinical rationale for the implementation should be established. Our findings imply that the integration of technology into busy working hours requires alignment with clinical workflow, incentives to justify the work, and organizational infrastructures, all crucial and underpinning factors for successful implementation and adaptation of eHealth portals in clinical care.

### Conclusion

The findings of this study show that by providing an eHealth portal to patients in a bariatric surgery program, health care professionals can observe patients’ writings and revelations, thereby capturing patient challenges and acting and implementing measures. Interacting with patients through the portal can prevent dropouts and patients’ health deterioration, factors that predict the success of the surgery. However, professionals report on organizational challenges and personal constraints related to communicating in writing with patients online. Further guidelines and education of professionals about how to handle, prioritize, communicate, and facilitate patients online is required, in addition to increased attention to organizational infrastructures, incentives, and rationales for enabling eHealth solutions in health care.

## Abbreviations

COPD: chronic obstructive pulmonary disease
PIN: personal identification number
SMS: short message service
